# CPIELA: Computational Prediction of Plant Protein–Protein Interactions by Ensemble Learning Approach From Protein Sequences and Evolutionary Information

**DOI:** 10.3389/fgene.2022.857839

**Published:** 2022-03-11

**Authors:** Li-Ping Li, Bo Zhang, Li Cheng

**Affiliations:** ^1^ College of Grassland and Environment Sciences, Xinjiang Agricultural University, Urumqi, China; ^2^ Xinjiang Key Laboratory of Grassland Resources and Ecology, Urumqi, China; ^3^ Xinjiang Technical Institute of Physics and Chemistry, Chinese Academy of Science, Urumqi, China

**Keywords:** plant, protein–protein interactions, machine learning, sequence, evolutionary information

## Abstract

Identification and characterization of plant protein–protein interactions (PPIs) are critical in elucidating the functions of proteins and molecular mechanisms in a plant cell. Although experimentally validated plant PPIs data have become increasingly available in diverse plant species, the high-throughput techniques are usually expensive and labor-intensive. With the incredibly valuable plant PPIs data accumulating in public databases, it is progressively important to propose computational approaches to facilitate the identification of possible PPIs. In this article, we propose an effective framework for predicting plant PPIs by combining the position-specific scoring matrix (PSSM), local optimal-oriented pattern (LOOP), and ensemble rotation forest (ROF) model. Specifically, the plant protein sequence is firstly transformed into the PSSM, in which the protein evolutionary information is perfectly preserved. Then, the local textural descriptor LOOP is employed to extract texture variation features from PSSM. Finally, the ROF classifier is adopted to infer the potential plant PPIs. The performance of CPIELA is evaluated via cross-validation on three plant PPIs datasets: *Arabidopsis thaliana*, *Zea mays*, and *Oryza sativa*. The experimental results demonstrate that the CPIELA method achieved the high average prediction accuracies of 98.63%, 98.09%, and 94.02%, respectively. To further verify the high performance of CPIELA, we also compared it with the other state-of-the-art methods on three gold standard datasets. The experimental results illustrate that CPIELA is efficient and reliable for predicting plant PPIs. It is anticipated that the CPIELA approach could become a useful tool for facilitating the identification of possible plant PPIs.

## Introduction

Plant protein–protein interactions (PPIs) participate in almost all aspects of cellular processes such as homeostasis control, signal transduction, organ formation, and plant defense (Morsy et al., 2008; Yuan et al., 2008; Fukao, 2012; Sheth and Thaker, 2014; Cheng et al., 2021). Thus, understanding plant PPIs could provide important insights into the pathological processes and the regulation of plant developmental processes. Consequently, constructing a PPI network at the system level is one of the key tasks to elucidate molecular mechanisms. In the past decades, several innovative high-throughput techniques, such as the yeast two-hybrid (Y2H) (Causier and Davies, 2002), bimolecular fluorescence complementation (BiFC) (Bracha-Drori et al., 2010), affinity purification coupled to mass spectrometry (AP-MS) (Puig et al., 2001), and protein microarrays (Hultschig et al., 2006), have been designed to detect plant PPIs. However, the aforementioned high throughput biological experiments have some unavoidable technical limitations (Yuan-Ke et al., 2019). For example, the number of PPIs obtained by high-throughput biological experiments is still much smaller than the number of expected PPIs (Aloy and Russell, 2004). It is believed that, for the most studied organisms (yeast), the number of PPIs is still underestimated (Sambourg and Thierry-Mieg, 2010). Furthermore, the techniques employed to detect plant PPIs are expensive and time-consuming, limiting the wide application of these approaches. In addition, most experimental techniques are often associated with high levels of a false-positive rate.

To conquer the disadvantages of previous biological approaches in a rapid and convenient way, computational approaches have become a hot research topic for predicting PPIs in proteomics research (Xiaoli et al., 2018; Lenz et al., 2020; He et al., 2021a; Green et al., 2021). In recent years, several public databases have been constructed to store the plant PPIs detected by biological experiments. For example, Dreze et al. constructed a proteome-wide binary PPI network of *Arabidopsis thaliana* consisting of more than 6,000 highly reliable PPIs among about 2,700 proteins (Dreze et al., 2011). Over the past decades, several computational methods that predict PPIs have been proposed by exploiting features ranging from network topology, protein sequence, phylogenetic profile, protein domain, and function annotation, among others (You et al., 2016a; Yi et al., 2018; Liu et al., 2019; Li et al., 2021). Min et al. generated a high-confident database of plant PPIs derived from the published studies and several databases (Min et al., 2010). Ding et al. used domain and ortholog identification combination approach to infer the genome-wide protein–protein interactions for *Citrus sinensis* (Ding et al., 2014). Geisler-Lee et al. presented a PPI network for *Arabidopsis thaliana*, predicted from interacting orthologs in *Caenorhabditis elegans*, *Saccharomyces cerevisiae*, *Homo sapiens*, and *Drosophila melanogaster* (Geisler-Lee et al., 2007). In another work by Brandao et al., a user-friendly tool, AtPIN, aggregated information on PPIs of *Arabidopsis thaliana*, sub-cellular localization, and ontology to map PPIs in *Arabidopsis thaliana* (Brandão et al., 2009). Zhu et al. constructed a genome-scale PPI network named PRIN in *Oryza sativa* by employing the InParanoid method based on the interolog approach. The PRIN approach integrated more than 533,000 PPIs among about 48,150 proteins from six organisms and detected more than 76,500 predicted rice PPIs among about 5,050 proteins (Zhu et al., 2011).

This work introduces a novel sequence-based computational approach, CPIELA, to predict potential plant protein–protein interactions. More specifically, we first converted the plant protein sequence into a position-specific scoring matrix (PSSM). Then, to fully capture the evolutionary information of the plant protein, we performed the local optimal-oriented pattern (LOOP) on the PSSM to extract the local textural descriptor. Although the LOOP algorithm is widely applied in image processing, to the best of our knowledge, this is the first work where LOOP is used in plant biology to predict PPIs. Finally, an efficient and powerful classification model, rotation forest (ROF), is used to identify the possible plant PPIs. The main contributions of this methodology are as follows: 1) based on the evolutionary history of proteins, the proposed method extracts the evolutionary features from the PSSM of the protein with known sequences, enabling our method to have more power for predicting plant PPIs than other sequence-based algorithms; 2) the proposed method does not depend on known PPIs samples and does not bias toward specific subspaces in the examined proteomic space because it directly captures features from the PSSMs of the plant protein sequence; and 3) we applied the ensemble ROF classifier to infer potential plant PPIs, which can truly improve the predictive accuracy compared with existing approaches. The proposed CPIELA method is well investigated on three plant PPIs datasets (*Arabidopsis thaliana*, *Zea mays*, and *Oryza sativa*) and yields high average accuracies of 98.63%, 98.09%, and 94.02%, respectively. In order to further verify the predictive performance of CPIELA, we compare it with the popular support vector machine (SVM) and random forest (RF) classifier. The experimental results illustrated that the CPIELA could be a complementary tool for plant PPIs prediction.

## Results and Discussions

### Evaluation Measures

**FIGURE 1:**
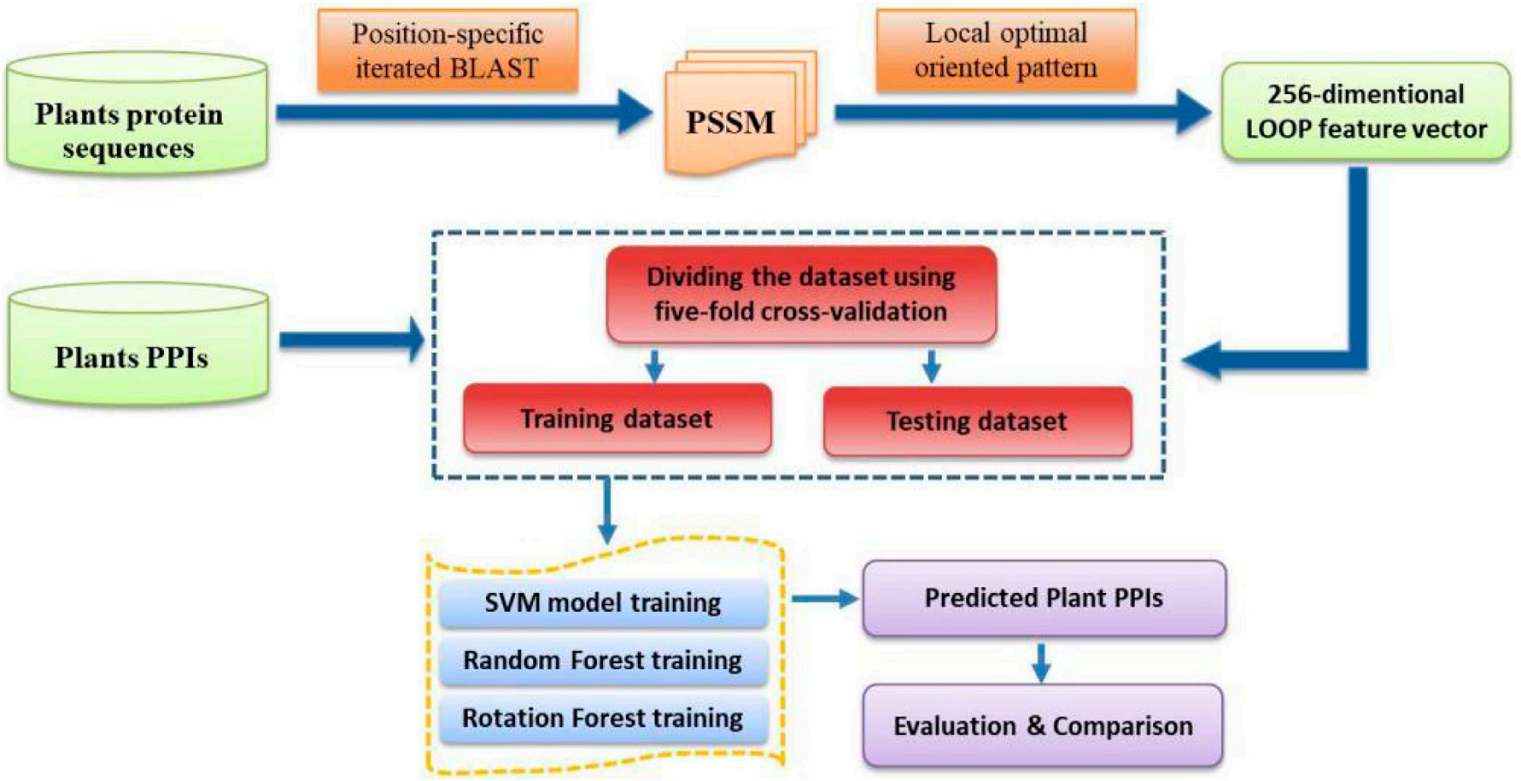
The flowchart of the proposed CPIELA method.

In the experiment, the fivefold cross-validation technique is used to evaluate the predictive performance of the CPIELA model. Cross-validation is a widely used approach to estimate the generalization performance of the prediction model. The *k*-fold cross-validation method usually randomly separates the instances into *k* equal-sized disjoint groups. Then, the *k*-1 groups are used as a training dataset, and the remaining group is retained as the testing samples. This process is repeated *k* times. The predictive results of the proposed method are evaluated using five criteria, including precision (Prec.), accuracy (Acc.), sensitivity (Sen.), specificity (Spec.), and Matthews correlation coefficient (MCC). The calculation formulas are listed as follows:

$$Accu.=\frac{TN+TP}{FP+FN+TP+TN},$$ (1)

$$Sen.=\frac{TP}{FN+TP},$$ (2)

$$Prec.=\frac{TP}{FP+TP},$$ (3)

$$Spec.=\frac{TN}{FP+TN},$$ (4)

$$MCC=\frac{TP\times TN-FP\times FN}{\sqrt{\left(TP+FP\right)\left(TN+FP\right)\left(TP+FN\right)\left(TN+FN\right)}},$$ (5)

where *TP*, *FP*, *TN*, and *FN* represent the number of true-positive, false-positive, true-negative, and false-negative samples, respectively. Furthermore, the Receiver Operating Characteristic (ROC) curve is employed to describe and compare the performance of a prediction model (Broadhurst and Kell, 2006). The *y*-axis and *x*-axis of the ROC curve are the sensitivity (the true positive rate, TPR) and 1 − specificity (the false positive rate, FPR), respectively. The area under the ROC curve (AUC) is a frequently used measure of performance for classification. An AUC of 0.5 means a random classifier, while the ideal value of AUC would be 1.0. For the convenience of presentation, the specific steps of the CPIELA method for identifying plant PPIs are shown in Figure 1.

### Evaluation of Model Predictive Ability

To verify the high predictive performance of the CPIELA model, we performed it on three plant PPIs datasets: *Arabidopsis thaliana*, *Oryza sativa*, and *Zea mays*. To guarantee the stability of the predictive results, the fivefold cross-validation technique is used to estimate the generalization capacity of the proposed learning model. Because the predictive performance of a rotation forest (ROF) ensemble is highly associated with the number *L* of decision trees (DT) and the number *K* of feature subset, a grid search method is conducted for tuning multiple parameters of the RF model. Considering the tradeoff between the computational complexity and accuracy rate, we set the number of decision trees to 3 and the number of feature subsets to 10 for all experiments.

The experimental results on the *Arabidopsis thaliana* dataset are outlined in Table 1. It can be seen from Table 1 that the average accuracy of the proposed method is as high as 98.63%. In order to further quantify the prediction performance of the proposed method, some other evaluation measures are calculated. From Table 1, we can observe that the overall sensitivity, precision, specificity, MCC, and AUC are 97.56%, 99.69%, 99.70%, 97.30%, and 0.9954, respectively. The standard deviations of them are 0.43%, 0.10%, 0.09%, 0.42%, and 0.0009, respectively.

**TABLE 1:** The fivefold cross-validation results achieved on the *A. thaliana* dataset using the proposed CPIELA method.

Testing set	Accu. (%)	Sen. (%)	Prec. (%)	Spec. (%)	MCC (%)	AUC
1	98.43	97.23	99.56	99.58	96.90	0.9957
2	98.78	97.99	99.61	99.60	97.59	0.9961
3	98.39	97.04	99.76	99.77	96.83	0.9936
4	98.89	97.98	99.76	99.77	97.80	0.9957
5	98.67	97.58	99.76	99.77	97.37	0.9956
Average	**98.63 ± 0.22**	**97.56 ± 0.43**	**99.69 ± 0.10**	**99.70 ± 0.09**	**97.30 ± 0.42**	**0.9954**

The bold values in these Tables mean the highest value in every column.

For the *Zea mays* dataset, it can be observed from Table 2 that the proposed CPIELA achieved good performance of accuracy 98.09%, precision 99.03%, sensitivity 97.13%, specificity 99.05%, MCC 96.25%, and AUC 0.9912, respectively. We also tested the CPIELA method on the *Oryza sativa* dataset. Table 3 lists the predictive results of fivefold cross-validation. We achieved the high accuracy of 94.02%, the precision value of 94.39%, the sensitivity value of 93.63%, the specificity value of 94.43%, the MCC value of 88.79%, and the AUC value of 0.9581 on the *Oryza sativa* dataset. Furthermore, from Table 3, we can also see that the standard deviations of accuracy, precision, sensitivity, specificity, MCC, and AUC are 1.45%, 2.20%, 1.08%, 2.19%, 2.61%, and 0.014, respectively.

**TABLE 2:** The fivefold cross-validation results achieved on the *Zea mays* dataset using the proposed CPIELA method.

Testing set	Accu. (%)	Sen. (%)	Prec. (%)	Spec. (%)	MCC (%)	AUC
1	97.82	96.59	99.07	99.08	95.74	0.9914
2	98.28	97.34	99.22	99.22	96.62	0.992
3	97.98	97.05	98.89	98.91	96.04	0.9902
4	98.00	97.00	98.91	98.96	96.07	0.9893
5	98.37	97.65	99.07	99.09	96.79	0.9931
Average	**98.09 ± 0.23**	**97.13 ± 0.40**	**99.03 ± 0.14**	**99.05 ± 0.12**	**96.25 ± 0.44**	**0.9912**

The bold values in these Tables mean the highest value in every column.

**TABLE 3:** The fivefold cross-validation results achieved on the *Oryza sativa* dataset using the proposed CPIELA method.

Testing set	Accu. (%)	Sen. (%)	Prec. (%)	Spec. (%)	MCC (%)	AUC
1	93.70	93.74	93.45	93.65	88.19	0.9558
2	93.59	92.17	95.17	95.09	88.00	0.9516
3	93.33	93.54	93.15	93.13	87.56	0.952
4	96.56	95.21	97.86	97.91	93.36	0.9826
5	92.92	93.49	92.32	92.36	86.84	0.9484
Average	**94.02 ± 1.45**	**93.63 ± 1.08**	**94.39 ± 2.20**	**94.43 ± 2.19**	**88.79 ± 2.61**	**0.9581**

The bold values in these Tables mean the highest value in every column.

**FIGURE 2:**
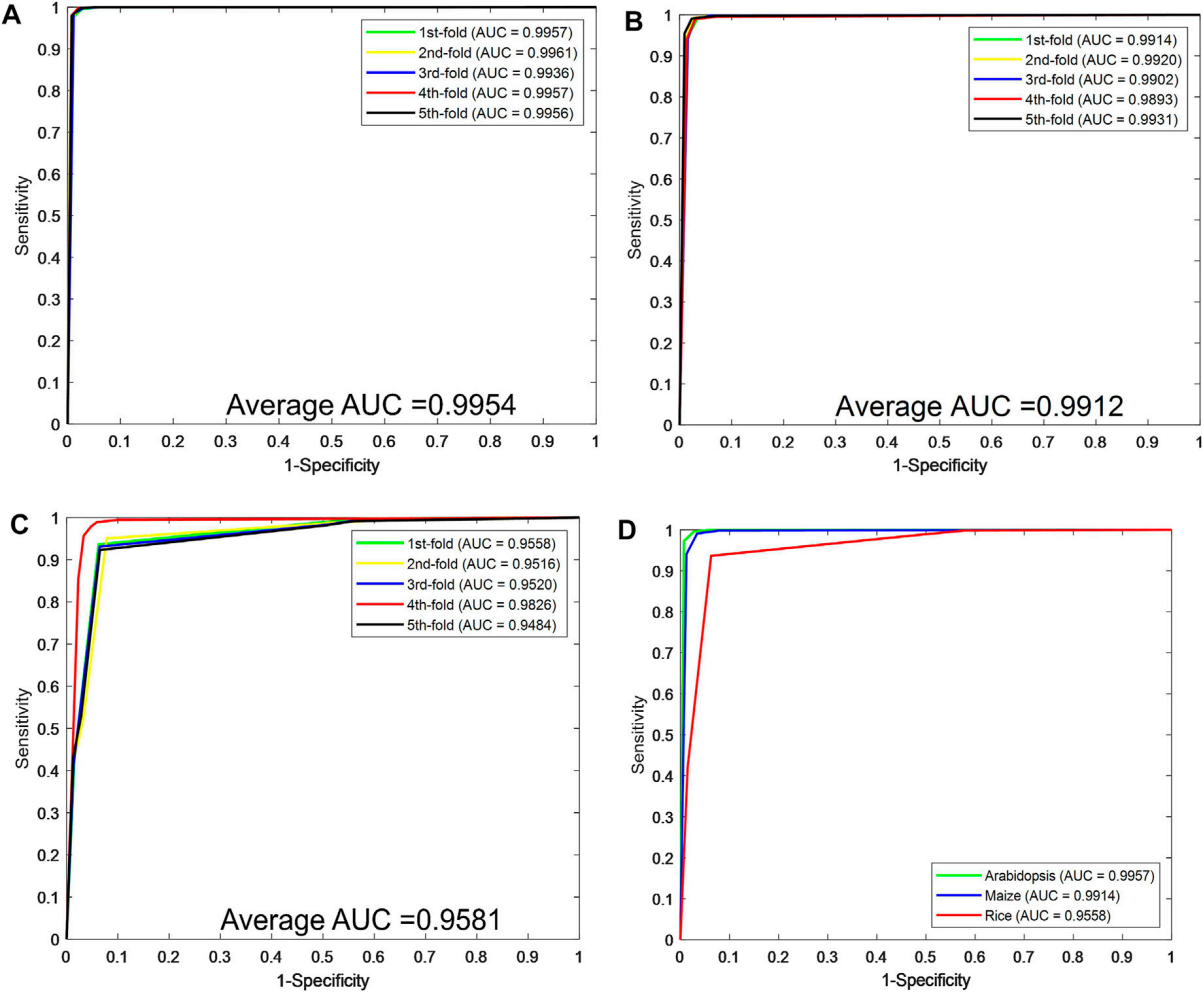
The predictive performance of the proposed CPIELA method via fivefold cross-validation. **(A–C)** The Receiver Operating Characteristic (ROC) curves of *Arabidopsis thaliana*, *Zea mays*, and *Oryza sativa* datasets. **(D)** The ROC curves performed by the CPIELA method on three plant PPIs datasets.

Figures 2A–C plot the ROC curves generated by the CPIELA method on the *Arabidopsis thaliana*, *Zea mays*, and *Oryza sativa* datasets. It can be seen from the above experimental results that the CPIELA method is effective for predicting plant PPIs. The better prediction performance mainly comes from the discriminative LOOP descriptors and the powerful ROF classifier. More specifically, the PSSM not only encodes the sequence into the matrix but also obtains sufficient evolutionary information on plant proteins, which can significantly improve the prediction accuracy. As a popular ensemble classifier, the ROF model has a considerably high predictive capability for identifying potential PPIs, making us more convinced that the proposed CPIELA can be a useful tool for predicting plant PPIs.

### Comparison of the Proposed Model With Different Classifiers and Descriptors

In this section, we conduct an experiment to compare the prediction performance of the state-of-the-art SVM classifier (Chih-Chung and Chih-Jen, 2011), the standard random forest (RF), and the rotation forest (ROF). The experimental results of the above-mentioned classifiers combined with the LOOP descriptor are listed in Table 4. It can be seen from Table 4 that the average accuracies of SVM, RF, and ROF classifier on the *Arabidopsis thaliana* dataset are 89.37%, 97.21%, and 98.63%, respectively. To demonstrate the predictive ability of the proposed CPIELA more comprehensively, we also computed the values of sensitivity, precision, MCC, and AUC. As observed from Table 4, the proposed CPIELA model achieved the highest performance on the *Arabidopsis thaliana* dataset with the sensitivity value of 97.56%, precision value of 99.69%, MCC value of 97.30%, and AUC value of 0.9954. In addition, we could observe in detail from Table 4 that the corresponding standard deviation of accuracy, precision, sensitivity, MCC, and AUC is 0.22%, 0.10%, 0.43%, 0.42%, and 0.0009, respectively.

**TABLE 4:** The fivefold cross-validation results achieved by different classifiers on the three plant datasets.

Dataset	Classifier	Acc. (%)	Sen. (%)	Prec. (%)	MCC (%)	AUC
*A. thaliana*	SVM	89.37 ± 0.25	83.95 ± 0.51	94.16 ± 0.41	80.89 ± 0.39	0.9495 ± 0.0038
	RF	97.21 ± 0.12	96.15 ± 0.19	98.22 ± 0.33	94.58 ± 0.22	0.9720 ± 0.0011
	Our method	**98.63 ± 0.22**	**97.56 ± 0.43**	**99.69 ± 0.10**	**97.30 ± 0.42**	**0.9954 ± 0.0009**
*Zea mays*	SVM	84.46 ± 0.20	77.55 ± 0.94	89.98 ± 0.47	73.5 ± 0.34	0.9179 ± 0.0048
	RF	94.65 ± 0.60	94.28 ± 0.66	94.98 ± 0.81	89.87 ± 1.07	0.9472 ± 0.0060
	Our method	**98.09 ± 0.23**	**97.13 ± 0.40**	**99.03 ± 0.14**	**96.25 ± 0.44**	**0.9912 ± 0.0015**
*Oryza sativa*	SVM	88.95 ± 1.44	83.23 ± 2.52	94.00 ± 0.72	80.24 ± 2.28	0.9445 ± 0.0068
	RF	90.90 ± 1.30	90.45 ± 1.58	91.29 ± 2.10	83.47 ± 2.11	0.9113 ± 0.0122
	Our method	**94.02 ± 1.45**	**93.63 ± 1.08**	**94.39 ± 2.20**	**88.79 ± 2.61**	**0.9581 ± 0.0140**

The bold values in these Tables mean the highest value in every column.

The precision, sensitivity, MCC, and AUC of the SVM classifier are 94.16%, 83.95%, 80.89%, and 0.9495, respectively. The precision, sensitivity, MCC, and AUC of the RF model are 98.22%, 96.15%, 94.58%, and 0.9720, respectively. It is evident that the SVM model achieved poor accuracy compared to the RF and ROF classifiers. It is specifically notable in the case of MCC. The proposed CPIELA method is the model with the best predictive results in terms of MCC for *Arabidopsis thaliana* PPIs datasets.

We also pay attention to the other two plant PPIs datasets. Table 4 shows the experimental results obtain on the *Zea mays* dataset, from which we can observe that the average accuracies of SVM, RF, and ROF classifiers are 84.46%, 94.65%, and 98.09%, respectively. Here, it could also be observed that the average accuracies obtained by the SVM, RF, and ROF models on the *Oryza sativa* dataset are 88.95%, 90.90%, and 94.02%, respectively.

**FIGURE 3:**
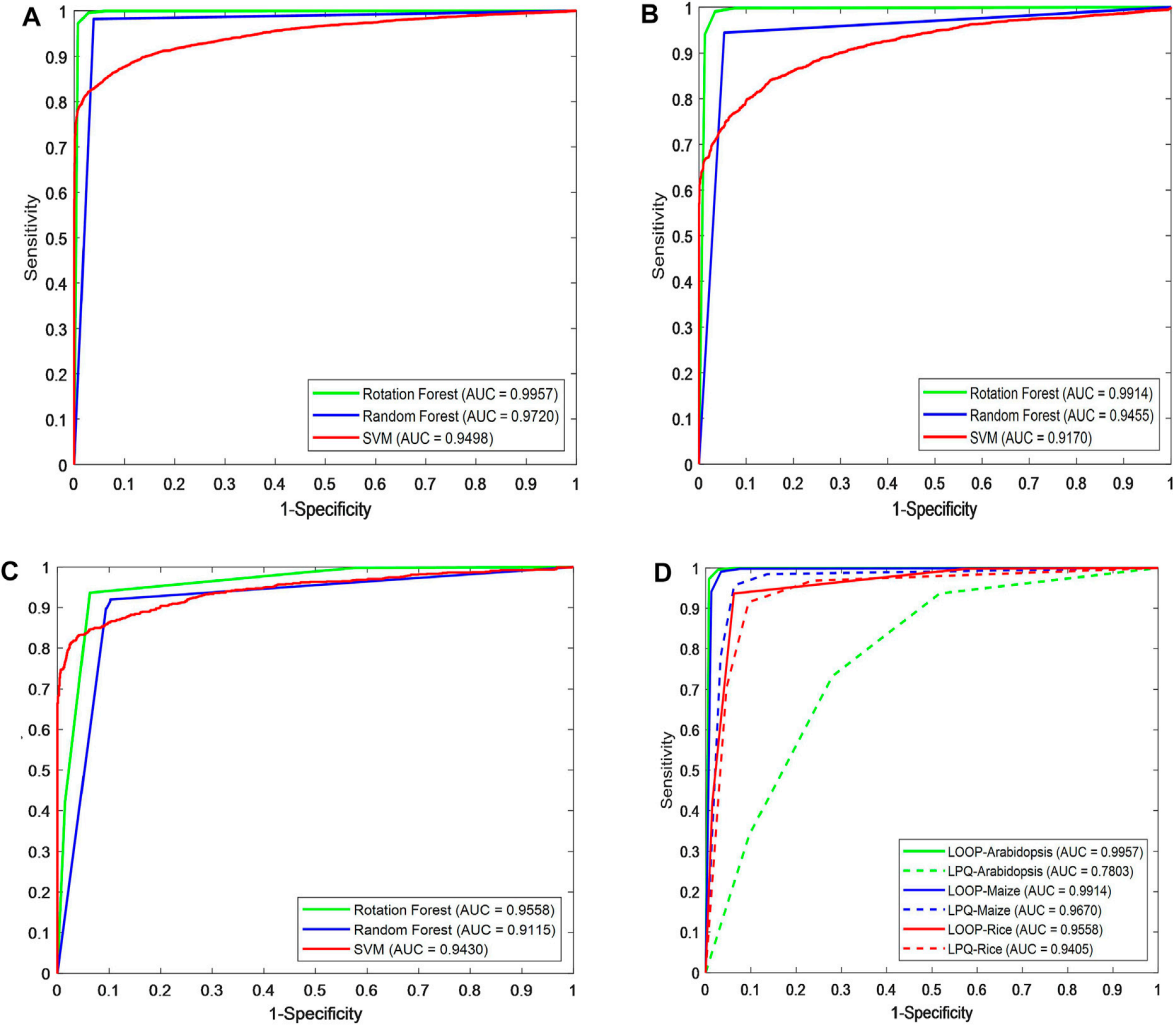
Prediction performance comparison of different classifiers using ROC curves in predicting plant protein–protein interactions. Shown in the plot are the ROC curves for **(A)** *Arabidopsis thaliana*, **(B)** *Zea mays*, **(C)** *Oryza sativa* datasets using RF (blue line), ROF (green line), SVM (red line), respectively. **(D)** ROC curves of different descriptors on three plant PPIs datasets.

Figures 3A–C show the ROC curve generated by different classifiers with the LOOP descriptor on the *Arabidopsis thaliana*, *Zea mays*, and *Oryza sativa* PPIs datasets, respectively.

In order to further evaluate the predictive performance of CPIELA, we also compared it with several other protein descriptors. In the experiment, local phase quantization (LPQ), first proposed by Ojansivu et al. (2008), Heikkilä et al. (2014), is employed to evaluate the performance of predicting plant PPIs on *Arabidopsis thaliana*, *Zea mays*, and *Oryza sativa* datasets, respectively. The fivefold cross-validation results of the LOOP and LPQ descriptor combined with ROF classifier on three plant PPIs datasets are summarized in Table 5. It can be observed that the LPQ descriptor achieved 73.17% average accuracy, 72.55% average sensitivity, 73.46% average precision, 73.79% average specificity, 60.74% average MCC, and 0.7873 average AUC on the *Arabidopsis thaliana* dataset. Meanwhile, the LOOP descriptor achieved 98.63% average accuracy, 97.56% average sensitivity, 99.69% average precision, 99.70% average specificity, 97.30% average MCC, and 0.9954 average AUC on the *Arabidopsis thaliana* dataset.

**TABLE 5:** The fivefold cross-validation results achieved on the three plant PPIs dataset among different descriptors using the proposed method.

Dataset	Methods	Acc. (%)	Sen. (%)	Prec. (%)	Spec. (%)	MCC (%)	AUC
*A. thaliana*	LPQ + RoF	73.17 ± 0.72	72.55 ± 0.86	73.46 ± 0.84	73.79 ± 0.64	60.74 ± 0.69	0.7873 ± 0.0090
	LOOP + RoF	**98.63 ± 0.22**	**97.56 ± 0.43**	**99.69 ± 0.10**	**99.70 ± 0.09**	**97.30 ± 0.42**	**0.9954 ±** **0.0009**
*Zea mays*	LPQ + RoF	94.17 ± 0.40	93.4 ± 0.64	94.86 ± 0.53	94.93 ± 0.50	89.02 ± 0.72	0.9639 ± 0.0031
	LOOP + RoF	**98.09 ± 0.23**	**97.13 ± 0.40**	**99.03 ± 0.14**	**99.05 ± 0.12**	**96.25 ± 0.44**	**0.9912 ± 0.0015**
*Oryza sativa*	LPQ + RoF	91.89 ± 0.64	92.14 ± 1.57	91.70 ± 0.87	91.65 ± 1.01	85.09 ± 1.07	0.9474 ± 0.0041
	LOOP + RoF	**94.02 ± 1.45**	**93.63 ± 1.08**	**94.39 ± 2.20**	**94.43 ± 2.19**	**88.79 ± 2.61**	**0.9581 ± 0.0140**

The bold values in these Tables mean the highest value in every column.

As we can see in Figure 3D, for *Arabidopsis thaliana*, the area under the ROC curve corresponding to LOOP is significantly larger than that of the LPQ descriptor. In terms of the indicator AUC, the AUC value of LOOP reaches 0.9957, which is 26.42% higher than that of the LPQ method. The experimental results also demonstrate that the LOOP descriptor exhibited significantly better performance than the LPQ descriptor on the other two plant PPIs datasets. Furthermore, the higher prediction accuracies and lower standard deviations indicate that the LOOP descriptor can effectively extract the features from protein sequence and significantly improve the predictive performance in plant PPIs prediction.

### Comparison With Existing Method

In the previous works, some researchers have put forward several computational approaches to solve the problem of plant PPIs prediction (Pan et al., 2021a; Pan et al., 2021b). Therefore, we compare the predictive performance of CPIELA against the recently proposed approaches. Experimental results of predictive performance comparison on *Oryza sativa* dataset between CPIELA and several related models are demonstrated in Table 6. It can be clearly observed from this table that the range of AUC generated by other approaches is from 0.7931 to 0.9440, the range of MCC obtained is from 37.39% to 78.26%, the range of accuracy generated by other models is from 66.63% to 82.60%, and the corresponding values obtained by CPIELA are 0.9581, 88.79%, and 94.02%. It shows that the predictive performance (AUC, MCC, accuracy) of CPIELA is better than that of existing models. We can see from Table 6 that the CPIELA model also gives better performance than the above-mentioned models for sensitivity, precision, and specificity metrics. Overall, the proposed CPIELA model shows better predictive performance than the previous prediction model on the *Oryza sativa* dataset.

**TABLE 6:** The predictive performance comparison of different methods on the *Oryza sativa* dataset.

Methods	Accu. (%)	Sen. (%)	Prec. (%)	Spec. (%)	MCC (%)	AUC
**DHT + KNN**	N/A	89.28 ± 0.78	76.41 ± 1.55	72.44 ± 1.58	68.59 ± 1.17	0.8680 ± 0.8900
**DHT + RF**	N/A	88.00 ± 1.34	87.30 ± 1.35	87.22 ± 1.16	78.26 ± 1.28	0.9199 ± 0.5800
**DHT + DNN**	82.60 ± 1.79	95.89 ± 0.91	75.79 ± 2.43	69.31 ± 3.53	67.65 ± 2.98	0.9440 ± 0.5800
**FFT + DNN**	75.31 ± 1.37	93.34 ± 1.59	68.61 ± 1.03	57.23 ± 2.90	54.26 ± 2.81	0.8760 ± 0.0096
**DWT + DNN**	81.54 ± 3.05	94.81 ± 0.65	75.10 ± 3.84	68.26 ± 6.61	65.50 ± 4.99	0.9309 ± 0.0052
**AC + DNN**	66.63 ± 4.48	88.42 ± 4.77	62.02 ± 4.91	45.02 ± 12.49	37.39 ± 5.39	0.7931 ± 0.0126
**DCT + DNN**	80.95 ± 1.10	96.12 ± 1.15	73.70 ± 1.41	65.64 ± 2.40	64.99 ± 1.97	0.9360 ± 0.0017
Our method	**94.02 ± 1.45**	**93.63 ± 1.08**	**94.39 ± 2.20**	**94.43 ± 2.19**	**88.79 ± 2.61**	**0.9581 ± 0.0140**

DHT: discrete Hilbert transform (Cizek, 1970); KNN: k-nearest neighbors; RF: random forest; FFT: fast Fourier transform; DWT: discrete wavelet transform; AC: auto covariance; DCT: discrete cosine transform.

The bold values in these Tables mean the highest value in every column.

## Conclusion

Protein–protein interactions are involved in almost all aspects of plant cellular processes. Thus, identifying plant PPIs is an important step toward understanding the molecular mechanisms and biological systems. This article developed a novel computational approach called CPIELA for predicting plant PPIs using the specifically designed protein representation method LOOP and ROF-based framework. The local optimal-oriented pattern (LOOP) descriptor is proposed to conquer some of the disadvantages in the previous feature descriptor, local directional pattern (LDP), and local binary pattern (LBP), by integrating the strength of these two descriptors. Thus, the LOOP-based features from PSSM are useful for predictive accuracy improvement. A highly accurate rotation forest algorithm is used to predict the potential plant PPIs. Experimental results on three plant PPIs datasets showed that the proposed CPIELA method outperforms all existing methods, demonstrating the feasibility and effectiveness of the proposed protein representation LOOP and the ROF-based classifier for predicting plant PPIs. The proposed sequence-based prediction method enables the systematic identification of possible PPIs in plants.

## Materials and Methodology

### Golden Standard Datasets

With the rapid advances of high-throughput biological technologies, many resources currently provide plant PPIs for different species. To construct a plant PPIs prediction model and compare it with existing prediction approaches, three plant PPIs datasets (*Zea mays*, *Oryza sativa*, and *Arabidopsis thaliana*) are employed in this work. For the interactome of *Zea mays*, 14,230 experimentally verified PPIs are downloaded from the Protein-Protein Interaction Database for Maize (PPIM) (Zhu et al., 2017) and agriGO (Tian et al., 2017). Because there is no available confirmed non-interacting plant PPIs, constructing negative PPIs dataset remains a challenging task in PPIs prediction. In order to build the negative dataset, 14,230 maize protein pairs located in different subcellular localization are randomly chose in this study. Consequently, the whole *Zea mays* dataset consists of 28,460 protein pairs.

A total of 4,800 non-redundant *Oryza sativa* protein interaction pairs among 1,834 rice proteins are downloaded from the PRIN database (http://bis.zju.edu.cn/prin) (Gu et al., 2011). The *Arabidopsis thaliana* PPIs dataset is collected from the public databases of BioGrid (Rose et al., 2018), TAIR (Yon et al., 2003), and IntAct (Kerrien et al., 2011). Meanwhile, the protein pairs containing a protein with fewer than fifty amino acids or having ≤40% sequence identity are removed. Finally, the 28,110 protein pairs from 7,437 *Arabidopsis thaliana* proteins comprise the positive dataset. The 28,110 protein pairs occurring in two different subcellular localizations are generated as a negative PPIs dataset. In this way, the whole *Arabidopsis thaliana* dataset is constructed by more than 56,220 protein pairs. The summary of plant PPIs used in this study is shown in Table 7.

**TABLE 7:** Summary of plant PPIs and proteins in different species.

Species name	Common name	Number of proteins	Number of PPIs
***Arabidopsis thaliana***	Thale cress	7, 437	56, 220
***Zea mays***	Maize	4, 841	28, 460
***Oryza sativa***	Rice	1, 834	9, 600

### Position-Specific Scoring Matrix

The position-specific scoring matrix (PSSM) was first proposed by Gribskov et al. to detect distantly related proteins and is now widely applied for the representation and prediction of PPIs (Gribskov et al., 1987; You et al., 2014; Wong et al., 2015; You et al., 2016b). A PSSM for a given protein is a 20×*M* matrix $P=\left\{P_{ij}:i=1,2,\ldots ,20\;and\;j=1,2,\ldots ,M\right\}$ , where *M* is the length of the target protein sequence. The PSSM matrix *p* can be represented as follows:

$$P=\left[\begin{array}{c} \begin{array}{cc} P_{1,1} & P_{1,2} \end{array}\begin{array}{cc} \;\;\;\cdots & P_{1,M} \end{array}\\ \begin{array}{cc} P_{2,1} & P_{2,2} \end{array}\begin{array}{cc} \;\;\;\cdots & P_{2,M} \end{array}\\ \begin{array}{cc} \vdots \;\;\;\;\;\; & \vdots \;\;\;\;\;\;\; \end{array}\begin{array}{cc} \vdots \;\;\;\; & \vdots \;\; \end{array}\\ \begin{array}{cc} P_{20,1} & P_{20,2} \end{array}\begin{array}{cc} \;\;\;\cdots & P_{20,M} \end{array} \end{array}\right],$$ (6)

where each element denotes the log-likelihood of the particular amino acid substitution at that position in the template. For example, it assigns a value $P_{i,j}$ for the *i*th residue in the *j*th position of the query protein sequence with a small score representing a weekly conserved position and a large score indicating a highly conserved position.

In the experiment, we employed the position-specific iterated BLAST (PSI-BLAST) tool and SwissProt database to build the PSSM for each protein amino acid sequence (Altschul et al., 1997; Altschul and Koonin, 1998; Amos and Rolf, 1999). The PSI-BLAST approach is highly sensitive in discovering similar proteins in distantly related species and new members of the protein family. To obtain high homologous sequences, we set the number of iterations to three, the e-value to 0.001, and the default value to the other parameters. The PSI-BLAST tool was downloaded from http://blast.ncbi.nlm.nih.gov/Blast.cgi.

### Local Optimal-Oriented Pattern

Tapabrata et al. presented the local optimal-oriented pattern (LOOP) as a novel binary local pattern descriptor that encodes rotation invariance into the main formulation of the local binary descriptor (Chakraborti et al., 2018). The LOOP descriptor is an improvement designed on local binary pattern (LBP) (Ojala et al., 1994) and local directional pattern (LDP) (Jabid et al., 2010).

**FIGURE 4:**
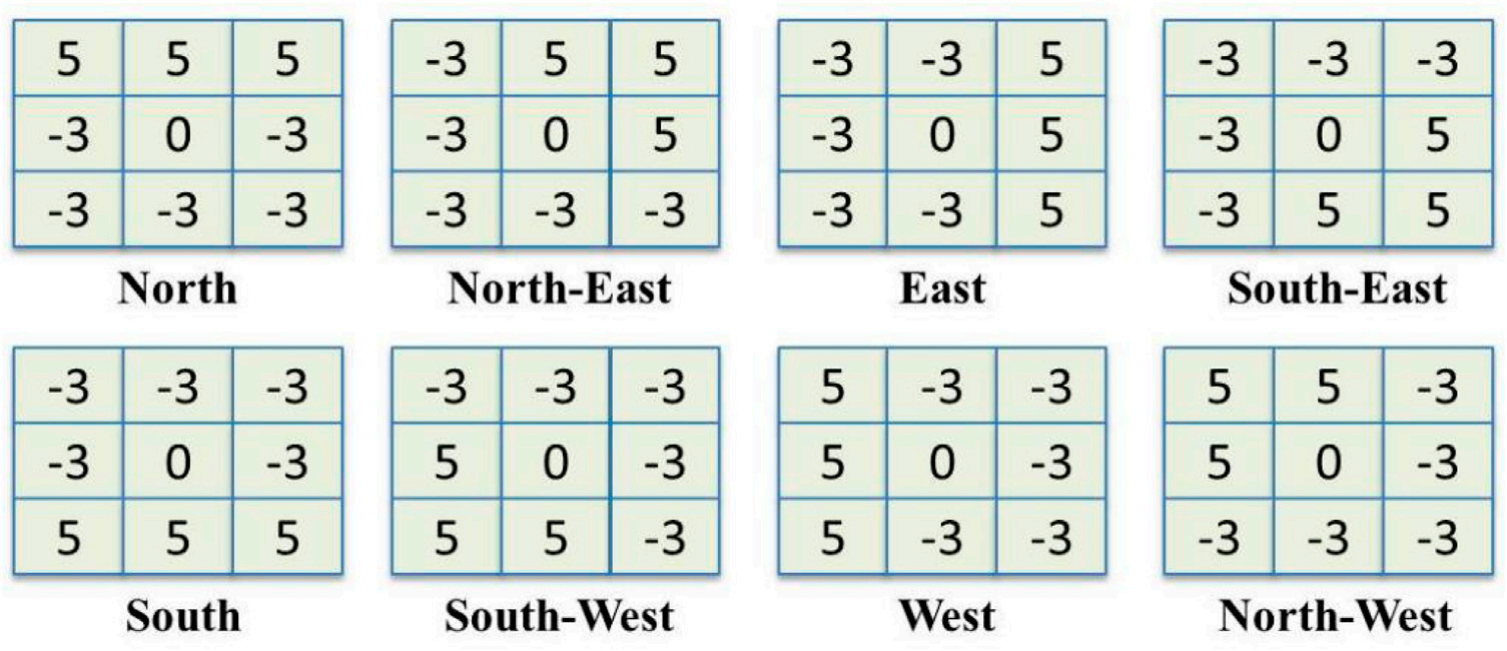
The masks of Kirsch’s edge detector which is used for calculating responses in eight possible directions.

Given an image I , let $i_{c}$ be the intensity at pixel $\left({\text{x}}_{c},y_{c}\right)$ . Suppose $i_{n}\;\left(n=0,\;1,\ldots ,\;7\right)$ represents the intensity of a pixel in the 3×3 neighborhood of $\left({\text{x}}_{c},y_{c}\right)$ keeping out the pixel $i_{c}$ . Figure 4 shows the Kirsch edge detectors centered at $\left({\text{x}}_{c},y_{c}\right)$ in eight directions. Let $m_{n}\;\left(n=0,\;1,\ldots ,\;7\right)$ be the eight responses of the Kirsch masks, corresponding to pixels with intensity $i_{n}\;\left(n=0,\;1,\ldots ,\;7\right)$ . Suppose $m_{k}$ is the *k*th highest Kirsch activation. An exponential $\omega_{n}$ for each of these pixels is assigned based on the rank of the magnitude of $m_{n}$ amongst the eight Kirsch mask outputs. Finally, the value of LOOP for the pixel $\left({\text{x}}_{c},y_{c}\right)$ is calculated as follows:

$$\text{LOOP}\left(x_{c},y_{c}\right)=\overset{7}{\underset{n=0}{\sum }}s\left(i_{n}-i_{c}\right).2^{\omega_{n}},$$ (7)

where

$$s\left(x\right)=\{\begin{array}{c} 1\;\;\;\;\;\;\;\;\;\;if\;x\geq 0\\ 0\;\;\;\;\;\;otherwise \end{array}.$$ (8)

where $i_{c}$ denotes the intensity of the center pixel (x,y) . In our study, the input PSSM is a 20×*M* matrix. Thus, each protein sequence is represented by a 256-dimensional feature vector after employing the LOOP descriptor.

### Rotation Forest

Rotation forest (ROF) is a popular ensemble classifier firstly proposed by Rodriguez et al. (2006). Compared with other classifiers, the ROF model is successfully used in dealing with many computational biology problems (He et al., 2021b). The basic idea of the rotation forest model is to simultaneously improve both individual accuracy and member diversity within an ensemble classifier. The success of the ROF method is attributed to the base classifier and rotation matrix created by the transformation algorithms, including principal component analysis (PCA) (Jolliffe, 2002), local fisher discriminant analysis (LFDA) (Masashi et al., 2010), maximum noise fraction (MNF) (Gordon, 2000), and independent component analysis (ICA) (Prasad, 2001). The framework of the ROF model is described as follows.

Let X be the training samples in the form of an N×n matrix, where *N* represents the number of samples and n denotes the number of features, respectively. Let a vector $Y={\left[y_{1},\ldots ,y_{N}\right]}^{T}$ be the corresponding class label, where $y_{j}\in \left\{\omega_{1},\ldots ,\omega_{c}\right\}$ . Let *F* be the feature set, and *F* is randomly split into *K* equal subset. Suppose *L* is the number of base decision trees in the ensemble model, which could be represented as ${\text{Γ}}_{1},{\text{Γ}}_{2},\ldots ,{\text{Γ}}_{L}$ , respectively. It should be noticed that the number of base classifiers (*L*) and the number of feature subsets (*K*) are the two important tuning parameters for the ROF classifier. The training dataset for a single classifier ${\text{Γ}}_{i}$ is preprocessed as follows:

1) Randomly divide *F* into *K* disjointed feature sets, each subset containing M=n/K features.
2) Let ${\text{F}}_{i,j}$ be the jth (j=1, 2,…,K) feature subset for the training dataset of classifier ${\text{Γ}}_{i}$ , and a new matrix ${\text{X}}_{i,j}$ is built by selecting the corresponding column of the features in the subset ${\text{F}}_{i,j}$ from the training dataset X . Then, a bootstrap subset of objects is selected with the size of 75 percent of the dataset ${\text{X}}_{i,j}$ to form a new training dataset $X_{i,j}^{'}$ .
3) The principal component analysis (PCA) technique is used on $X_{i,j}^{'}$ to obtain the coefficients in a matrix ${\text{C}}_{i,j}$ .
4) A sparse rotation matrix ${\text{R}}_{i}$ is constructed using the coefficients obtained in the matrix ${\text{C}}_{i,j}$ , which is expressed as follows:

$$R_{i}=\left[\begin{array}{ccc} a_{i,1}^{\left(1\right)},\ldots ,a_{i,1}^{\left(M_{1}\right)} & 0 & \begin{array}{cc} \ldots & 0 \end{array}\\ 0 & a_{i,2}^{\left(1\right)},\ldots ,a_{i,2}^{\left(M_{2}\right)} & \begin{array}{cc} \ldots & 0 \end{array}\\ \begin{array}{c} \vdots \\ 0 \end{array} & \begin{array}{c} \vdots \\ 0 \end{array} & \begin{array}{cc} \begin{array}{c} \ddots \\ \ldots \end{array} & \begin{array}{c} \vdots \\ a_{i,K}^{\left(1\right)},\ldots ,a_{i,K}^{\left(M_{K}\right)} \end{array} \end{array} \end{array}\right].$$ (9)

The columns of ${\text{R}}_{i}$ should be rearranged to $R_{i}^{a}$ according to the original feature set. Then, the transformed training dataset for classifier ${\text{Γ}}_{i}$ will become $XR_{i}^{a}$ . In this way, all classifiers are trained in parallel.

In the prediction phase, provided a testing sample x , let $d_{i,k}\left(xR_{i}^{a}\right)$ be the probability generated by the classifier ${\text{Γ}}_{i}$ to the hypothesis that x belongs to class $\omega_{k}$ . Then, the confidence of each class is calculated by means of the average combination as follows:

$$\mu_{k}\left(x\right)=\frac{1}{L}\overset{L}{\underset{i=1}{\sum }}d_{i,k}\left(xR_{i}^{a}\right),\;\;\;k=1,\ldots ,c.$$ (10)

Finally, the testing sample x is assigned to the class with the largest confidence.

## Data Availability

The original contributions presented in the study are included in the article/Supplementary Material, further inquiries can be directed to the corresponding authors.

## References

[B1] Aloy P. Russell R. B. (2004). Ten Thousand Interactions for the Molecular Biologist. Nat. Biotechnol. 22 (10), 1317–21. 10.1038/nbt1018 15470473

[B2] Altschul S. F. Koonin E. V. (1998). Iterated Profile Searches with PSI-BLAST-A Tool for Discovery in Protein Databases. Trends Biochem. Sci. 23 (11), 444–447. 10.1016/s0968-0004(98)01298-5 9852764

[B3] Altschul S. Madden T. L. Schffer A. A. Zhang J. Zhang Z. Webb M. (1997). Gapped BLAST and PSI-BLAST: a New Generation of Protein Database Search Programs. Nucleic Acids Res. 25 (17), 3389–3402. 10.1093/nar/25.17.3389 9254694 PMC146917

[B4] Amos B. Rolf A. (1999). The SWISS-PROT Protein Sequence Data Bank and its Supplement TrEMBL in 1999. Nucleic Acids Res. (1), 49. 9847139 10.1093/nar/27.1.49 PMC148094

[B5] Bracha-Drori K. Shichrur K. Katz A. Oliva M. Angelovici R. Yalovsky S. (2010). Detection of Protein-Protein Interactions in Plants Using Bimolecular Fluorescence Complementation. Plant J. 40 (3), 419–427. 10.1111/j.1365-313X.2004.02206.x 15469499

[B6] Brandão M. Dantas L. L. Silva-Filho M. C. (2009). AtPIN: *Arabidopsis thaliana* Protein Interaction Network. Bmc Bioinformatics 10, 454. 10.1186/1471-2105-10-454 20043823 PMC2810305

[B7] Broadhurst D. I. Kell D. B. (2006). Statistical Strategies for Avoiding False Discoveries in Metabolomics and Related Experiments. Metabolomics 2 (4), 171–196. 10.1007/s11306-006-0037-z

[B8] Causier B. Davies B. (2002). Analysing Protein-Protein Interactions with the Yeast Two-Hybrid System. Plant Mol. Biol. 50 (6), 855–870. 10.1023/a:1021214007897 12516858

[B9] Chakraborti T. McCane B. Mills S. Pal U. (2018). LOOP Descriptor: Local Optimal-Oriented Pattern. IEEE Signal. Process. Lett. 25, 635–639. 10.1109/lsp.2018.2817176

[B10] Cheng F. Zhao J. Wang Y. Lu W. Loscalzo J. (2021). Comprehensive Characterization of Protein–Protein Interactions Perturbed by Disease Mutations. Nat. Genet. 53 (3), 1–12. 10.1038/s41588-020-00774-y 33558758 PMC8237108

[B11] Chih-Chung C. Chih-Jen L. (2011). Libsvm: A Library for Support Vector Machines.

[B12] Cizek V. (1970). Discrete Hilbert Transform. IEEE Trans. Audio Electroacoust. 18 (4), 340–343. 10.1109/tau.1970.1162139

[B13] Ding Y.-D. Chang J.-W. Guo J. Chen D. Li S. Xu Q. (2014). Prediction and Functional Analysis of the Sweet orange Protein-Protein Interaction Network. BMC Plant Biol. 14 (1), 213. 10.1186/s12870-014-0213-7 25091279 PMC4236729

[B14] Dreze M. Carvunis A-R. Charloteaux B. Galli M. (2011). Evidence for Network Evolution in an Arabidopsis Interactome Map. Science 333 (6042), 601–607. 10.1126/science.1203877 21798944 PMC3170756

[B15] Fukao Y. (2012). Protein-protein Interactions in Plants. Plant Cel Physiol. 53 (4), 617–625. 10.1093/pcp/pcs026 22383626

[B16] Geisler-Lee J. O'Toole N. Ammar R. Provart N. J. Millar A. H. Geisler M. (2007). A Predicted Interactome for Arabidopsis. Plant Physiol. 145 (2), 317–329. 10.1104/pp.107.103465 17675552 PMC2048726

[B17] Gordon C. (2000). A Generalization of the Maximum Noise Fraction Transform. IEEE Trans. Geosci. Remote Sensing 38 (1), 608–610. 10.1109/36.823955

[B18] Green A. G. Elhabashy H. Brock K. P. Maddamsetti R. Kohlbacher O. Marks D. S. (2021). Large-scale Discovery of Protein Interactions at Residue Resolution Using Co-evolution Calculated from Genomic Sequences. Nat. Commun. 12 (1), 1396. 10.1038/s41467-021-21636-z 33654096 PMC7925567

[B19] Gribskov M. McLachlan A. D. Eisenberg D. (1987). Profile Analysis: Detection of Distantly Related Proteins. Proc. Natl. Acad. Sci. 84 (13), 4355–4358. 10.1073/pnas.84.13.4355 3474607 PMC305087

[B20] Gu H. Zhu P. Jiao Y. Meng Y. Chen M. (2011). PRIN: a Predicted rice Interactome Network. Bmc Bioinformatics 12, 161. 10.1186/1471-2105-12-161 21575196 PMC3118165

[B21] He T. Bai L. Ong Y. S. (2021b). Vicinal Vertex Allocation for Matrix Factorization in Networks. IEEE T Cybern (99). Piscataway, NJ: IEEE (The Institute of Electrical and Electronics Engineers). 10.1109/tcyb.2021.3051606 33600331

[B22] He T. Ong Y. S. Bai L. (2021a). Learning Conjoint Attentions for Graph Neural Nets. San Diego, CA: NIPS; The Neural Information Processing Systems (NIPS) Foundation.

[B23] Heikkilä J. Rahtu E. Ojansivu V. (2014). Local Phase Quantization for Blur Insensitive Texture Description. Stud. Comput. Intelligence 506, 49–84. 10.1007/978-3-642-39289-4_3

[B24] Hultschig C. Kreutzberger J. Seitz H. Konthur Z. Bussow K. Lehrach H. (2006). Recent Advances of Protein Microarrays. Curr. Opin. Chem. Biol. 10 (1), 4–10. 10.1016/j.cbpa.2005.12.011 16376134 PMC7108394

[B25] Jabid T. Kabir M. H. Chae O. (2010). Gender Classification Using Local Directional Pattern (LDP).in” 20th International Conference on Pattern Recognition, ICPR 2010, 23-26. 10.1109/icpr.2010.373

[B26] Jolliffe I. T. (2002). Principal Component Analysis. J. Marketing Res. 87 (4), 513.

[B27] Kerrien S. Aranda B. Breuza L. Bridge A. Broackes-Carter F. Chen C. (2011). The IntAct Molecular Interaction Database in 2012. Nucleic Acids Res. 40 (Database issue), D841–D846. 10.1093/nar/gkr1088 22121220 PMC3245075

[B28] Lenz S. Sinn L. R. O'Reilly F. J. Fischer L. Rappsilber J. (2020). Reliable Identification of Protein-Protein Interactions by Crosslinking Mass Spectrometry. London: Nature Publishing Group. 10.1038/s41467-021-23666-z PMC8196013 34117231

[B29] Li H.-L. Pang Y.-H. Liu B. : BioSeq-BLM: a Platform for Analyzing DNA, RNA and Protein Sequences Based on Biological Language Models. 2021, 49(22):e129. 10.1093/nar/gkab829 PMC8682797 34581805

[B30] Liu B. Gao X. Zhang H. (2019). BioSeq-Analysis2.0: an Updated Platform for Analyzing DNA, RNA and Protein Sequences at Sequence Level and Residue Level Based on Machine Learning Approaches. Nucleic Acids Res. 47, e127. 10.1093/nar/gkz740 31504851 PMC6847461

[B31] Masashi W. Sugiyama T. Idé S. NakajimaJun (2010). Semi-supervised Local Fisher Discriminant Analysis for Dimensionality Reduction. Mach Learn.

[B32] Min M. Cai H. Zheng W. Yang Z. Feng Q. (2010). “A Database of Protein-Protein Interactions in Plants,” in International Conference on Bioinformatics & Biomedical Engineering, Wuhan, China, May 10–12, 2011.

[B33] Morsy M. Gouthu S. OrchardHarper S. Thorneycroft D. Harper J. F. Mittler R. (2008). Charting Plant Interactomes: Possibilities and Challenges. Trends Plant Sci. 13 (4), 183–191. 10.1016/j.tplants.2008.01.006 18329319

[B34] Ojala T. Pietikainen M. , and Harwood D. (1994). Performance Evaluation of Texture Measures with Classification Based on Kullback Discrimination of Distributions.IEEE

[B35] Ojansivu V. Rahtu E. Heikkila¨ J. (2008). Rotation Invariant Local Phase Quantization for Blur Insensitive Texture Analysis.”in 19th International Conference on Pattern Recognition. 10.1109/icpr.2008.4761377

[B36] Pan J. Li L-P. You Z-H. Yu C-Q. Ren Z-H. Guan Y-J. (2021a). Prediction of Protein–Protein Interactions in Arabidopsis, Maize, and Rice by Combining Deep Neural Network with Discrete Hilbert Transform. Front. Genet. 2021 (1678), 12. 10.3389/fgene.2021.745228 PMC8488469 34616437

[B37] Pan J. Li L-P. Yu C-Q. You Z-H. Ren Z-H. Tang J-Y. (2021b). A Novel Computational Approach to Predict Plant Protein-Protein Interactions via an Ensemble Learning Method. Scientific Programming 2021, 1607946. 10.1155/2021/1607946

[B38] Prasad P. S. (2001). Independent Component Analysis. Cambridge University Press.

[B39] Puig O. Caspary F. Rigaut G. Rutz B. Bouveret E. Bragado-Nilsson E. (2001). The Tandem Affinity Purification (TAP) Method: A General Procedure of Protein Complex Purification. Methods 24 (3), 218–229. 10.1006/meth.2001.1183 11403571

[B40] Rodriguez J. J. Kuncheva L. I. Alonso C. J. (2006). Rotation forest: A New Classifier Ensemble Method. IEEE Trans. Pattern Anal. Mach. Intell. 28 (10), 1619–1630. 10.1109/tpami.2006.211 16986543

[B41] Rose O. Chris S. Bobby-Joe B. Jennifer R. Lorrie B. Christie C. (2018). The BioGRID Interaction Database: 2019 Update. Nucleic Acids Res. 47, D529–D541. 10.1093/nar/gky1079 PMC6324058 30476227

[B42] Sambourg L. Thierry-Mieg N. (2010). New Insights into Protein-Protein Interaction Data lead to Increased Estimates of the *S. cerevisiae* Interactome Size. Bmc Bioinformatics 11 (1), 605. 10.1186/1471-2105-11-605 21176124 PMC3023808

[B43] Sheth B. P. Thaker V. S. (2014). Plant Systems Biology: Insights, Advances and Challenges. Planta: Int. J. Plant Biol. 10.1007/s00425-014-2059-5 24671625

[B44] Tian T. Liu Y. Yan H. You Q. Yi X. Du Z. (2017). agriGO v2.0: a GO Analysis Toolkit for the Agricultural Community, 2017 Update. Nucleic Acids Res. 45 (W1), W122–W129. 10.1093/nar/gkx382 28472432 PMC5793732

[B45] Wong L. You Z. H. Li S. Huang Y. A. Liu G. (2015). “Detection of Protein-Protein Interactions from Amino Acid Sequences Using a Rotation Forest Model with a Novel PR-LPQ Descriptor,” in International Conference on Intelligent Computing. 10.1007/978-3-319-22053-6_75

[B46] Xiaoli Q. Chen Z. Xiucai Y. Pu-Feng D. Ran S. Leyi W. (2018). CPPred-FL: a Sequence-Based Predictor for Large-Scale Identification of Cell-Penetrating Peptides by Feature Representation Learning. Brief. Bioinformatics. 10.1093/bib/bby091 30239616

[B47] Yi H. C. You Z. H. Huang D. S. Li X. Jiang T. H. Li L. P. (2018). A Deep Learning Framework for Robust and Accurate Prediction of ncRNA-Protein Interactions Using Evolutionary Information. Mol. Ther. Nucleic Acids 11 (C), 337–344. 10.1016/j.omtn.2018.03.001 29858068 PMC5992449

[B48] Yon R. S. William B. Berardini T. Z. Chen G. David D. Aisling D. (2003). The Arabidopsis Information Resource (TAIR): a Model Organism Database Providing a Centralized, Curated Gateway to Arabidopsis Biology, Research Materials and Community. Nucleic Acids Res. 31 (1), 224 12519987 10.1093/nar/gkg076 PMC165523

[B49] You Z-H. Li X. Chan K. C. (2016b). An Improved Sequence-Based Prediction Protocol for Protein-Protein Interactions Using Amino Acids Substitution Matrix and Rotation forest Ensemble Classifiers. Neurocomputing.

[B50] You Z. H. Li S. Gao X. Luo X. Ji Z. (2014). Large-Scale Protein-Protein Interactions Detection by Integrating Big Biosensing Data with Computational Model. Biomed. Res. Int. 2014, 598129. 10.1155/2014/598129 25215285 PMC4151593

[B51] You Z. H. Zhou M. C. Luo X. Li S. (2016a). Highly Efficient Framework for Predicting Interactions between Proteins. IEEE T Cybern. 10.1109/TCYB.2016.2524994 28113829

[B52] Yuan J. S. Galbraith D. W. Dai S. Y. Griffin P. Stewart C. N. (2008). Plant Systems Biology Comes of Age. Trends Plant Sci. 13 (4), 165–171. 10.1016/j.tplants.2008.02.003 18329321

[B53] Yuan-Ke Z. Zi-Ang S. Han Y. Tao L. Yang G. Pu-Feng D. (2019). Predicting lncRNA-Protein Interactions with miRNAs as Mediators in a Heterogeneous Network Model. Front. Genet. 10, 1341. 10.3389/fgene.2019.01341 32038709 PMC6988623

[B54] Zhu G. Rui J. Zhao X. M. (2017). PPIM: A Protein-Protein Interaction Database for Maize.in” 13th IEEE Conference on Automation Science and Engineering (IEEE CASE 2017), sponsored by the IEEE Robotics and Automation Society (RAS), Xi'an, China, 20–23 Auguest 2017. 10.1109/coase.2017.8256085

[B55] Zhu P. Gu H. Jiao Y. Huang D. Chen M. (2011). Computational Identification of Protein-Protein Interactions in Rice Based on the Predicted Rice Interactome Network. Genomics Proteomics Bioinformatics 9 (4), 128–137. 10.1016/S1672-0229(11)60016-8 22196356 PMC5054448

